# The Urinary Cytokine/Chemokine Signature of Renal Hyperfiltration in Adolescents with Type 1 Diabetes

**DOI:** 10.1371/journal.pone.0111131

**Published:** 2014-11-13

**Authors:** Ron L. H. Har, Heather N. Reich, James W. Scholey, Denis Daneman, David B. Dunger, Rahim Moineddin, R. Neil Dalton, Laura Motran, Yesmino Elia, Livia Deda, Masha Ostrovsky, Etienne B. Sochett, Farid H. Mahmud, David Z. I. Cherney

**Affiliations:** 1 Division of Nephrology, University Health Network - Toronto General Hospital, Banting and Best Diabetes Centre, University of Toronto, Toronto, Ontario, Canada; 2 Division of Nephrology, University Health Network - Toronto General Hospital, Toronto, Ontario, Canada; 3 Department of Pediatrics, Division of Endocrinology, Hospital for Sick Children, University of Toronto, Toronto, Ontario, Canada; 4 Department of Pediatrics, University of Cambridge, Cambridge, United Kingdom; 5 Family and Community Medicine, University of Toronto Toronto, Ontario, Canada; 6 WellChild Laboratory, Evelina Children's Hospital, St Thomas' Hospital, London, United Kingdom; University of San Francisco, United States of America

## Abstract

**Objective:**

Urinary cytokine/chemokine levels are elevated in adults with type 1 diabetes (T1D) exhibiting renal hyperfiltration. Whether this observation extends to adolescents with T1D remains unknown. Our first objective was to determine the relationship between hyperfiltration and urinary cytokines/chemokines in normotensive, normoalbuminuric adolescents with T1D using GFR_cystatin_. Our second aim was to determine the relationship between urine and plasma levels of inflammatory biomarkers, to clarify the origin of these factors.

**Methods:**

Urine and serum cytokines/chemokines (Luminex platform) and GFR_cystatin_ were measured in normofiltering (n = 111, T1D-N, GFR<135 ml/min/1.73 m^2^) and hyperfiltering (n = 31, T1D-H, GFR≥135 ml/min/1.73 m^2^) adolescents with T1D (ages 10–16), and in age and sex matched healthy control subjects (HC, n = 59).

**Results:**

We noted significant step-wise increases in urinary cytokine/chemokine excretion according to filtration status with highest levels in T1D-H, with parallel trends in serum analyte concentrations. After adjusting for serum glucose at the time of sampling, differences in urinary cytokine excretion were not statistically significant. Only serum IL-2 significantly differed between HC and T1D (p = 0.0076).

**Conclusions:**

Hyperfiltration is associated with increased urinary cytokine/chemokine excretion in T1D adolescents, and parallel trends in serum cytokine concentration. The GFR-associated trends in cytokine excretion may be driven by the effects of ambient hyperglycemia. The relationship between hyperfiltration, glycemia, and variations in serum and urine cytokine expression and their impact on future renal and systemic vascular complications requires further study.

## Introduction

Diabetes mellitus is the most common cause of end-stage renal failure requiring renal placement therapy in the developed world. Unfortunately, despite the use of renin angiotensin aldosterone system (RAAS) blockers and intensive glycemic control, a significant proportion of patients continue to develop diabetic nephropathy. Furthermore, in type 1 diabetes (T1D), RAAS inhibition-based primary prevention of diabetic nephropathy, defined by changes on renal biopsy, has been unsuccessful [1]. The failure of current therapies may in part be due to a clinical inability to distinguish high-risk patients from those who may never develop complications. It is therefore critical to identify the role of additional factors that contribute to the initiation and progression of diabetic nephropathy to guide more targeted treatment strategies.

Hyperglycemia is necessary for the development of diabetic nephropathy in experimental models and in humans [2], [3], [4]. In young adult patients with T1D, acute clamped hyperglycemia increases the urinary excretion of pro-inflammatory and pro-fibrotic factors implicated in the pathogenesis of diabetic nephropathy, including eotaxin, fibroblast growth factor-2, granulocyte-monocyte colony stimulating factor (GM-CSF), interferon-α2, interleukin (IL)-12, IL-2, monocyte chemoattractant protein-3 (MCP-3), MCP-1, macrophage-derived chemokine (MDC), macrophage inflammatory proteins-1α (MIP-1α), platelet derived growth factor-BB (PDGF-AB/BB), tumour necrosis factor-β and sCD40 Ligand (sCD40L) [5]. We have further shown that renal hyperfiltration is associated with higher levels of urinary cytokines/chemokines compared with T1D patients with normal GFR values (T1D-N) and healthy controls (HC) [6]. Moreover, the increase in urinary cytokine/chemokine excretion induced by hyperglycemia is blunted by RAAS inhibition, and this effect is exaggerated in patients with T1D and renal hyperfiltration (T1D-H) [7]. However, GFR in this previous work was measured directly by inulin clearances under clamped glycemic conditions, and these techniques can only be used in controlled research laboratory setting. It is not known if renal hyperfiltration, defined with clinically applicable cystatin C-based methods, is also associated with increased urinary cytokine/chemokine excretion in an ambulatory setting [8].

The identification of urinary biomarkers of preclinical kidney disease that could be used clinically to distinguish adolescents with T1D at increased risk of developing renal disease is an important research goal for clinicians who take care of similar patients in the pediatric setting and as these patients transition to adult care [9]. Accordingly, our aim was to determine the relationship between GFR and urinary cytokines/chemokines in normotensive, normoalbuminuric adolescents with T1D and normal renal function. We hypothesized that in an adolescent cohort, urinary cytokines/chemokines would be elevated in patients with T1D-H compared to both T1D-N and a similar group of age and sex matched healthy control participants.

## Research Design and Methods

### Study Population and Ethnics Statement

Patients were recruited from the longitudinal, observational, non-interventional arm of the Adolescent Type 1 Diabetes Cardio-Renal Intervention Trial (AdDIT), from clinical sites in the Greater Toronto Area. In brief, the Non-Randomized Low-Risk arm of AdDIT is a 4-year observational/natural history study, following adolescents at low and middle risk of developing microalbuminuria (EudraCT Number: 2007-001039-72). High-risk adolescents are recruited into the AdDIT Interventional Study (http://www.clinicaltrials.gov/ct2/show/NCT01581476), which was designed to examine the effect of angiotensin converting enzyme inhibitors and statins on renal, retinal and cardiovascular endpoints. Our study did not include participants involved in the intervention trial, however, as an ancillary component of Non-Randomized Low-Risk arm of AdDIT we also included high-risk subjects who chose not to enter the AdDIT Intervention Study. All analyses in this manuscript were performed using biological specimens and data collected from subjects enrolled in the observational and ancillary arm of AdDIT, specifically baseline data from the study obtained at Greater Toronto Area sites.

T1D patients were recruited from endocrinology clinics at The Hospital for Sick Children, Credit Valley Hospital and Markham-Stouffville in the Greater Toronto Area (Ontario, Canada), while healthy controls were recruited through local advertisements. The Hospital for Sick Children was the primary site of recruitment and also coordinated recruitment at the secondary sites (REB#: 1000012240). The Hospital for Sick Children Research Ethics Board, Credit Valley Hospital Ethics Forum and Markham-Stouffville Research Ethics Board approved the protocol and the consent procedure. In accordance to the Declaration of Helsinki, written consent and informed consent was obtained from the legal guardian/next of kin/caretakers of minors aged 15 and younger, while the minors provided assent. All subjects, aged 16 and older with capacity to understand the study information, gave complete written and informed consent to participate in the study.

Normotensive, normoalbuminuric participants with T1D were recruited. T1D patients were analyzed based on whether GFR was in the normal range (n = 111, GFR 90–134 ml/min/1.73 m^2^) or hyperfiltration range (n = 31, GFR≥135 ml/min/1.73 m^2^) according to Larsson method, as we have previously published [8], [10]. Fifty-nine healthy controls with normal renal function were also included for comparison.

Inclusion criteria for T1D patients were: age 10–16, duration of type 1 diabetes ≥1 year, no history of hypertension, proteinuria, renal disease or macrovascular disease. Two sets of 3 early morning urines were obtained and microalbuminuria was defined as an ACR>3.5 mg/mmol/l in males and >4.0 mg/mmol/l in females in 2 out of the 3 consecutive early morning urines [11]. In addition to those delineated in the AdDIT observational protocol, other exclusion criteria included chronic inflammatory disease, anti-inflammatory or corticosteroid medicines or medications that interfere with the renin angiotensin aldosterone system (RAAS) [12].

### Sample Collection and Analytical Methods

Participants were asked to provide a first morning urine sample. This 50 ml mid-stream sterile urine specimen was used to measure levels of cytokines/chemokines using an established Cytokine/Chemokine Panel Luminex Assay urine creatinine concentration [5], [9]. Immediately after collection, urine was centrifuged at 1500 g for 15 minutes to remove cells, then separated into 1 ml aliquots and frozen at −80°C. Urine was then thawed at 4°C one day prior to use. Due to the urine specimen handling protocol designed to avoid protein degradation, this analysis included spot urines and not the timed collections. Furthermore, *a priori* we only included selected analytes that either increase in response to hyperglycemia or renal hyperfiltration in our previous work in young adults [5], [6]. Our analysis therefore included eotaxin, FGF-2, GM-CSF, IFNα2, IL-2, IL-12, MCP-3, MCP-1, MDC, MIP-1α, TNFβ, sCD40L, PDGF-AB/BB. We limited our analysis to these factors to maintain statistical power, to minimize false positive results, and to further elucidate mechanisms that may link high intraglomerular pressure with factors that promote initiation of renal disease. The accuracy and precision of the urinary cytokine/chemokine assay is available through the vendor at http://www.millipore.com/userguides/tech1/proto_mpxhcyto-60k. The detection limits of our assays have also been published previously [9]. The investigator performing data analysis was blinded to all study parameters.

Serum cystatin C was measured by a single operator using thawed samples by an immunoassay (Dade Behring Diagnostics, Newark, DE, USA) conducted on a BN Prospec System nephelometer. The between-assay coefficient of variation in samples from the lowest and highest quartiles of the cystatin C distribution was 6.2 and 0.9%, respectively. Cystatin C based GFR was derived using the body-surface area corrected Larsson formula, as described previously, which has superior operating characterstics compared with creatinine-based measurements in the hyperfiltration range using GFR_INULIN_ [10], [13].

Urinary albumin to creatinine ratio was determined from a spot urine collection by immunoturbidimetry. Hemoglobin A1C (HbA1c) was measured by high-performance liquid chromatography [14].

### Statistical analysis

All analyses in the manuscript were based on specimens and data collected at the baseline visit. Descriptive statistics were used to describe the sample. The baseline clinical and demographic characteristics were compared using appropriate test statistics. Between-group differences were determined by ANOVA, adjusted for multiple comparisons using Bonferroni's correction. The statistical package SAS 9.3 (SAS Institute, Cary, NC, USA) was used to analyze the data. Our sample size calculation was based on differences in urinary MCP-1 excretion due to the consistent relationship between this cytokine and renal disease. [5], [6], [15] Our previous data have shown that the SD for MCP-1 is 17 units [5], [6], [15]. To have an 80% power to detect a significant 25 unit between-group difference in MCP-1, for a two- sided test with p = 0.01, the sample size should be ≥16 per group. Participants with diabetes were analyzed on the basis of filtration status determined using cystatin C as described previously [8], [10]. Filtration status was determined at the end of the study once cystatin C and creatinine assays were complete for the entire cohort. In the first analysis, *between-group* comparisons were adjusted for age, gender, ACR and HbA1c. In the second analysis, blood glucose at the time of the urine sample collection was included rather than HbA1c, since our previous work has demonstrated that acute, ambient glycemia increases urinary cytokine/chemokine excretion [5], [7]. Additional statistical corrections for height and weight were not made because these parameters are already accounted for in eGFR equations. Serum levels of cytokines/chemokines were similarly analyzed using both dichotomous and continuous methods, except that ACR was not included.

## Results

### Baseline characteristics

For baseline demographic and biochemical parameters, the T1D-N had a higher proportion of males (56%) compared to HC (44%) and T1D-H (40%), and significant differences in age, weight and height were present (Table 1). The three groups had a heterogeneous composition of ethnicities. As expected, SBP tended to be higher in T1D groups compared to HC [16] and ACR within the normal range was higher in the T1D groups vs. HC, but the same in T1-N vs. T1D-H. For T1D-N and T1D-H, HbA1c and diabetes duration values were similar.

**Table 1:** Clinical Characteristics and Biochemistry in Healthy Controls and in Patients with Type 1 Diabetes Mellitus with Normofiltration or Hyperfiltration According to GFRcystatin C (mean ± SD).

	Healthy Controls(n = 59)	Normofiltration diabetic group (n = 111)	Hyperfiltration diabetic group (n = 31)	p-value
*Clinical characteristics:*				
Male/female	26/33	63/48	9/22	0.017
Ethnicity – n (%)				
White (Causcasian)	31 (52.5)	73 (65.8)	10 (32.3)	0.0005
Black (African, Caribbean)	3 (5.1)	7 (6.3)	4 (12.9)	0.395
South Asian	6 (10.2)	9 (8.1)	3 (9.7)	0.279
South East Asian	12 (20.3)	6 (5.4)	4 (12.9)	0.247
Aboriginal	0	0	0	-
Other	7 (11.9)	16 (14.1)	10 (32.2)	0.240
Age (years)	13.9±2.0	14.5±1.6	15.0±1.6	0.024
Diabetes duration (years)	N/A	7.4±3.1	7.3±3.3	0.890
Weight (kg)	54.2±13.0	62.2±14.1	60.4±16.13	0.017
Height (cm)	162±12	165±10	160±10	0.0008
Systolic blood pressure (mmHg)	111±10	116±11	114±7	0.683
Diastolic blood pressure (mmHg)	68±7	67±7	70±7	0.359
Pulse (beats per minute)	49±6.5	52±9	49±8	0.109
*Biochemistry:*				
Blood glucose (mmol/L)	4.7±0.7	9.3±4.5	11.5±3.4	<0.0001
HbA1c (%)	5.4±0.3	8.4±1.2	8.5±1.1	<0.0001
HbA1c (mmol/mol)	31±0.06	75±0.15	75±0.15	
Urine albumin/creatinine ratio (mg/mmol)	0.60±0.45	1.14±2.46	1.17±1.36	0.013
Serum LDL (mmol/L)	2.38±0.76	2.29±0.76	2.24±0.51	0.773
Serum triglyceride (mmol/L)	0.92±0.40	0.84±0.40	0.85±0.26	0.293

p-values provided for blood pressure are for z-scores; Blood glucose value obtained at the time of urine collection.

### The effect of renal filtration status on urinary cytokine/chemokine excretion

**Figure 1:**
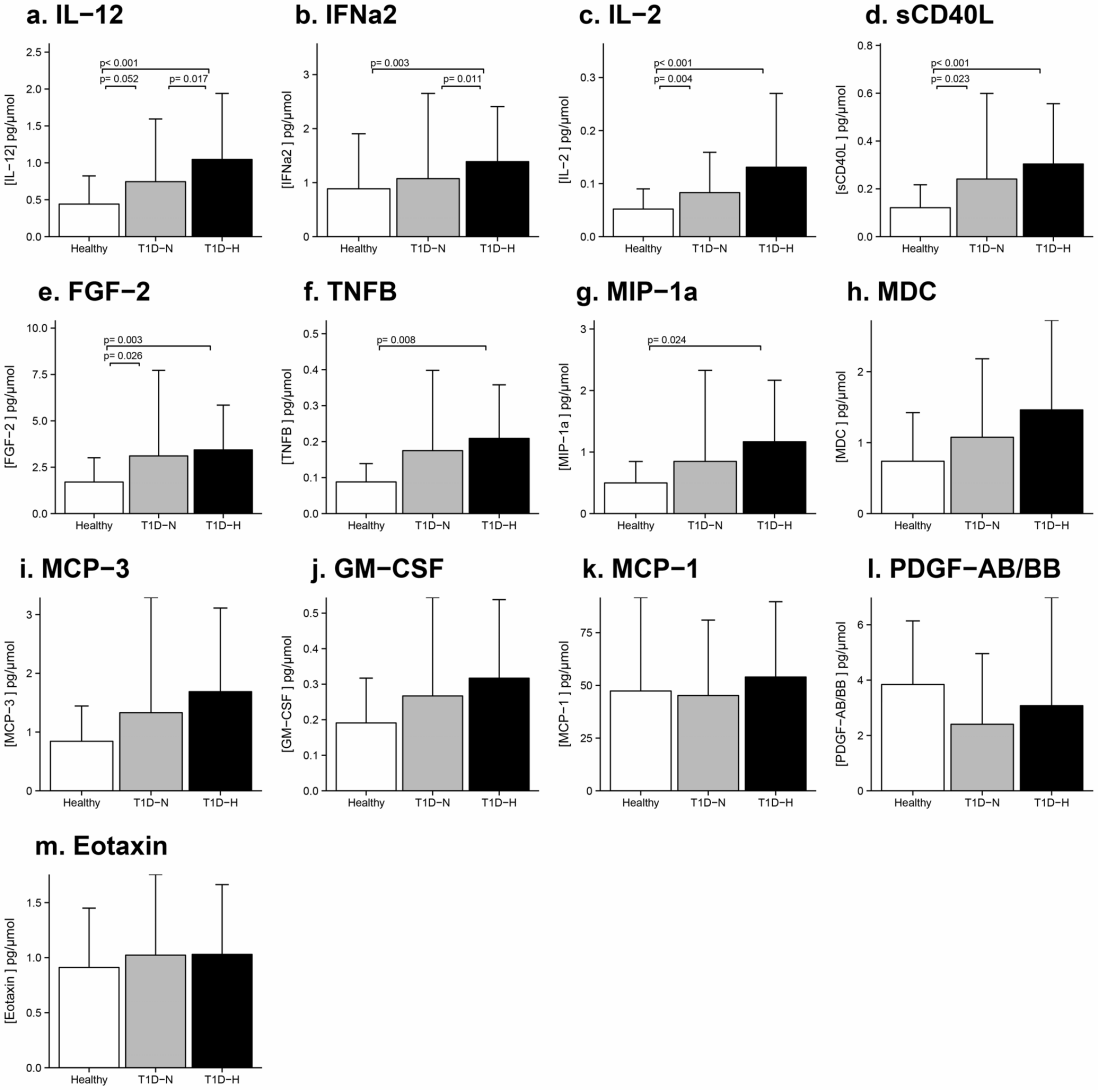
Urinary Excretion of Cytokine/Chemokines in Adolescents with Type 1 Diabetes According to Hyperfiltration Status vs Healthy Controls. Step-wise trends were observed for IL-12, IFNα2, IL-2, sCD40L, FGF-2, TNF-β, MIP-1α, MDC, MCP-3, GM-CSF, adjusted for age, gender, ACR and HbA1c. P-values show pair-wise comparisons with Bonferroni correction. After adjusting for plasma glucose at the time of collection, instead of HbA1c, pair-wise comparisons between normofilterers (T1D-N) and hyperfilterers (T1D-H) were no longer significant.

After adjusting for baseline clinical characteristics including HbA1c and ACR, the first pattern observed was a step-wise increase from HC to T1D-N to T1D-H for urinary IL-12 (Figure 1, panel a, ANOVA p = 0.0005). Pair-wise comparisons were also significant except for HC vs. T1D-N (p = 0.0518). For IFNα2 (Figure 1, panel b, ANOVA p = 0.0019), pair-wise differences between HC vs. T1D-H and T1D-N vs. T1D-H were significant, while differences in HC vs. T1D-N were not. Finally, levels of IL-2 (ANOVA p = 0.0002), sCD40L (ANOVA p = 0.001), FGF-2 (ANOVA p = 0.0038) (Figure 1, panels c–e), generally increased from HC to T1D-N to T1D-H, but only pairwise differences for HC vs. T1D-N and HC vs. T1D-H reached significance. For TNF-β (ANOVA p = 0.0097) and MIP-1α (ANOVA p = 0.0174) (Figure 1, panel f–g), only HC and T1D-H group differences were significant. Similar trends for MDC, MCP-3 and GM-CSF did not reach significance. When ethnicity was added as a covariable to the regression model, *between-group* differences in urinary cytokine/chemokine excretion persisted.

After adjusting for the same baseline clinical characteristics including plasma glucose rather than HbA1c in a regression analysis, *between-group* differences for IL-12 and IFNα2 were no longer significant.

### Correlation between urinary cytokines/chemokines and renal function, adjusted for age, gender, ACR and hemoglobin A1c

For HC, only PDGF-AB/BB (β = 0.0433, p = 0.0019) correlated with GFR_cystatin C_. In the T1D group, GFR_cystatin C_ correlated with MCP-1 (β = 0.3189, p = 0.0162) and PDGF-AB/BB (β = 0.0231, p = 0.0331).

### Serum levels of cytokines/chemokines in HC and T1D Groups

**Figure 2:**
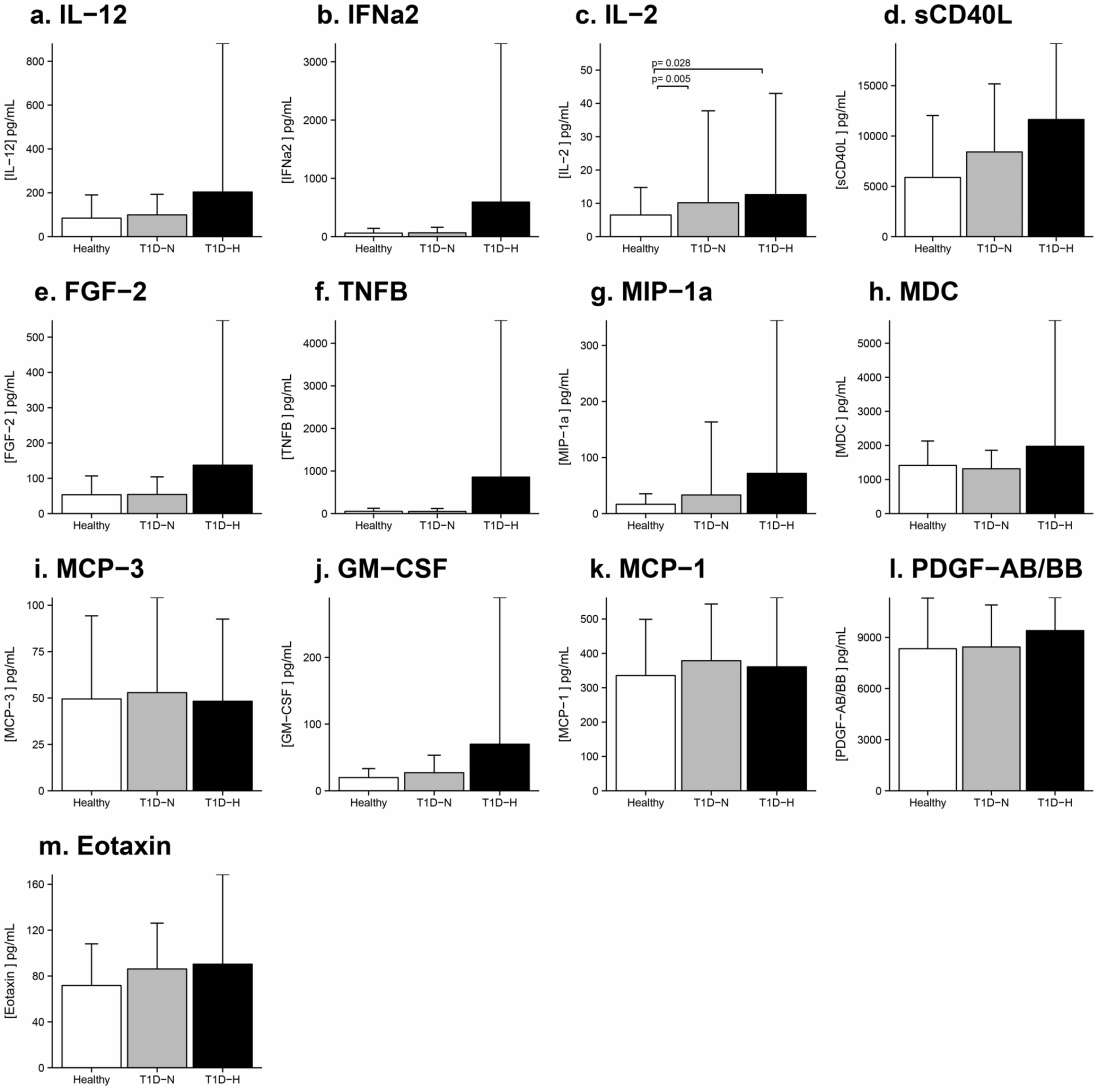
Serum Cytokine/Chemokine Signature in Adolescents with Type 1 Diabetes Based on Hyperfiltration Status and Healthy Controls. A parallel trend to urinary excretion of cytokine/chemokines was observed, although only IL-2 showed significance. P-values show pair-wise comparisons with Bonferroni correction, adjusted for age, gender and HbA1c.

For serum markers, *between-group* differences for IL-2 in HC vs. T1D-N and T1D-H reached significance (Figure 2), and the addition of ethnicity to the model had no effect. In the continuous analysis comparing serum analyte levels with GFR in the HC group, serum MIP-1α (β = −0.2752, p = 0.0384), MDC (β = −12.702, p = 0.0061), IL-12 (β = −2.1089, p = 0.0237) correlated with GFR_cystatin C_. In the T1D cohort, correlations were also observed between serum IL-12 (β = 4.0269, p = 0.0029), IFNα2 (β** = **15.8123, p = 0.0015), FGF-2 (β = 2.1275, p = 0.0032), TNF-β (β = 21.7215, p = 0.0028), MDC (β = 20.3889, p = 0.0018), GM-CSF (β = 1.1626, p = 0.0029), PDGF-AB/BB (β = 20.0651, p = 0.0232) and GFR_cystatin C_.

### Correlation between urine and serum levels of cytokines/chemokines in HC and T1D participants

In HC, none of the urine cytokines/chemokines correlated with corresponding cytokines/chemokines in serum. In the T1D group, correlations between urinary and plasma levels of eotaxin (r = 0.20, p = 0.02), sCD40L (r = 0.22, p = 0.009) and GM-CSF (r = 0.23, p = 0.0062) reached statistical significance.

## Discussion

Albuminuria is an early clinical marker of diabetic nephropathy. Unfortunately, albuminuria is limited as a predictive biomarker because many patients exhibit stable levels of albuminuria and never develop impaired renal function, and spontaneous regression of albuminuria is also common [17]. Furthermore, progressive early GFR decline without albuminuria is common [18] and may reflect underlying vascular disease and tubulointerstitial inflammation rather than traditional diabetic glomerulosclerosis [19], [20]. The identification of sensitive and specific early biomarkers to predict the development of nephropathy prior to the onset of albuminuria, such as urinary cytokines/chemokines, is therefore of the utmost importance.

Urinary cytokine/chemokine excretion is associated with factors that promote diabetic nephropathy, such as hyperglycemia, as well as hyperfiltration, a surrogate marker for high intraglomerular pressure leading to glomerulosclerosis [5], [6]. Despite these promising pathophysiological rationale for the use of cytokines/chemokines as markers of early renal disease, the role of these factors as markers of diabetic nephropathy prior to the onset of albuminuria remain unclear, especially in adolescents [21]. Our aim was therefore to determine the relationship between GFR and urinary cytokines/chemokines in a cohort of normotensive, normoalbuminuric adolescents with T1D analyzed on the basis of renal filtration status, since hyperfiltration may identify a group of individuals who are at an increased risk of developing clinical nephropathy in T1D and T2D [2], [4].

Our first major finding was that renal hyperfiltration was associated with higher levels of urinary cytokines/chemokines in T1D adolescents prior to the development of microalbuminuria. There were also parallel trends in serum cytokine concentrations. This panel of biomarkers was selected due to associations between these factors with 1) renal hyperfiltration and acute responses to clamped hyperglycemia and 2) renal injury leading to diabetic nephropathy, including chemotaxis, inflammation and fibrosis [5], [6] and 3) hyperglycemia-related increases in urinary excretion of cytokine/chemokines, including IFN, PDGF, TNF and MCP-1 [22], [23], [24], leading to chronic kidney disease [25], [26], [27], [28], [29]. In contrast with our previous work in adults using gold standard inulin clearance techniques to measure GFR under clamped euglycemic and hyperglycemic conditions, we used cystatin C-based measurements, since this estimate of GFR can be used clinically and has superior operating characteristics compared to creatinine-based estimates within the hyperfiltration range when compared to inulin clearances [8], [10], [30].

To account for the influence of ambient hyperglycemia at the time of GFR measurement on urinary cytokines/chemokines, we performed analyses with either HbA1c or plasma glucose as covariables [5]. Ambient plasma glucose concentration was an important determinant of urinary cytokine/chemokine excretion. On regression analysis, adjustment for serum glucose at the time of sampling mitigated the observed differences in cytokine excretion. This observation supports the hypothesis that ambient hyperglycemia is an independent determinant of urinary inflammatory biomarkers, and should be accounted for in future work. Chronic glycemic control, reflected by HbA1C, did not have the same confounding influence on cytokine excretion. This suggests that perhaps transient glycemia is more important than chronic glycemic status in determining acute variations in cytokine/chemokine production.

Renal hyperfiltration has been attributed to hyperglycemia-mediated activation of the RAAS causing efferent renal arteriolar vasoconstriction, and also to diabetes related increases in proximal tubular sodium-glucose cotransport, leading to altered tubuloglomerular feedback and afferent arteriolar vasodilatation [31], [32]. Therefore, hyperglycemia may induce renal inflammation through direct mitogenic, angiotensin II-dependent pathways, and may also act indirectly through neurohormonal and tubular mechanisms that raise intraglomerular pressure and wall tension, leading to pro-inflammatory effects [33], [34]. Regardless of the responsible mechanism, hyperfiltration is associated with the initiation and progression of nephropathy in T1D and T2D, and is present in 20–60% of young patients with T1D [2], [4], [35]. Mechanistically, high intraglomerular pressure is associated with hyperfiltration and increased wall tension in experimental diabetes, promoting renal parenchymal inflammation [36], [37]. To determine if a similar relationship exists in humans, we previously compared urinary cytokine/chemokine excretion in adult patients with T1D with or without hyperfiltration defined by inulin-based clearances under controlled laboratory conditions [5], [6]. We demonstrated that T1D-H exhibit higher urinary cytokines/chemokines levels vs. T1D-N and HC [6]. In a separate study we demonstrated that clamped hyperglycemia, a stimulus for hyperfiltration, also increases urinary cytokine/chemokine excretion [5]. To determine if hyperfiltration-related increases in urinary cytokines/chemokines are reversible, we examined the effect of RAAS blockade on these factors and demonstrated 1) a decline in many of these mediators and 2) that these effects were exaggerated hyperfilterers [7]. It was, however, unclear if these observations were applicable outside of a controlled laboratory setting, or if our observations would extend to an adolescent cohort. To our knowledge, the present study is the first to examine the interaction between renal hyperfiltration, the earliest known hemodynamic abnormality related to the development of diabetic nephropathy, and urinary markers of inflammation in adolescents in a clinical setting. These results therefore confirm our previous work in adults with T1D, and suggest that inflammation associated with hyperfiltration begins much earlier in the natural history of the disease, potentially identifying an opportunity for future primary prevention strategies.

To determine whether the increase in urinary cytokines/chemokines in T1D-H patients is due to high systemic levels resulting in renal “overflow” or instead due to more local production, we also measured serum levels of each factor. Our second major finding was that in contrast with many *between-group* differences for urinary factors, serum levels of IL-2 differed between the three groups, with highest levels in T1D-H. Although by no means definitive, this observation suggests an interaction between systemic and renal levels of IL-2, possibly reflecting systemic production and consequent renal clearance. Interestingly, urinary levels of IL-12, IFNα2, FGF-2 and TNF-β tended to increase from HC to T1D-N to T1D-H, and serum levels of these factors correlated with GFR, again suggesting a relationship between systemic and renal levels of these factors. Even in the cases of MDC and GM-CSF where *between-group* differences for urinary levels were not significant, serum levels of these factors tended to follow the same trend, with a positive correlation between serum levels of GFR. We and others have previous observed systemic hemodynamic abnormalities in T1D-H patients, including endothelial dysfunction and higher blood pressure, suggesting that hyperfiltration reflects a generalized abnormality of the endothelium and vasculature rather than an isolated renal abnormality [16], [38], [39]. Therefore, our findings suggest that at least for IL-2, IL-12, IFNα2, FGF-2 and TNF-β higher urinary excretion rates in T1D-H may have been on the basis of increased clearance from the systemic circulation rather than renal production and subsequent urinary excretion.

In addition to increased risk of developing albuminuria and GFR loss in clinical trials [2], [4], T1D patients with hyperfiltration including adolescents exhibit greater hemodynamic responses to ACE inhibition, reflected by declines in GFR toward the normal range [32]. We have also shown that T1D-H exhibit similar effects when tubuloglomerular feedback is activated using the sodium glucose cotransport inhibitor empagliflozin [40]. Finally, RAAS blockade results in greater urinary cytokine/chemokine suppression in T1D-H versus T1D-N [7]. Hyperfiltration therefore represents a distinct physiological state that identifies a subgroup of patients who may be at an increased risk of diabetic nephropathy, and who also exhibit greater hemodynamic and molecular responses to potential renal protective agents. Regardless of the source of cytokines/chemokines in the present study cohort, our results suggest that T1D-H patients generally exhibit higher levels of factors that have been linked with renal and cardiovascular injury. As such, patients with T1D-related hyperfiltration may therefore represent a high-risk group that should be targeted for earlier therapeutic interventions in future studies.

Our study has some important limitations. First, we were not able to study this large cohort under clamped glycemic conditions. We have demonstrated in previous work that strictly controlled physiologic environment that transient hyperglycemia significantly influences cytokine/chemokine levels. We therefore intentionally sought to study this in a more realistic clinical setting, and accounted for glycemia a priori with our analytic approach. A second limitation was the use of GFR based on estimating equations rather than direct GFR measures such as inulin. Nevertheless, use of GFR_cystatin C_ to define hyperfiltration has provided important insights into how urinary cytokine/chemokine excretion rates may be translated into the clinical setting in future work [8]. Third, in this analysis, we hypothesized that *between-group* differences in urinary cytokines/chemokines were primarily on the basis of systemic overflow, and that increased renal production was also possible. We recognize, however, that children with T1D commonly exhibit evidence of proximal tubular dysfunction [41], [42] and we have recently reported that proximal tubular function may be different in T1D-H vs. T1D-N [31]. As such, future work should determine if physiological differences in proximal tubule function, rather than activation of inflammatory pathways, contribute to abnormalities in urinary cytokine/chemokine handling, resulting in step-wise changes from HC to T1D-N to T1D-H observed in this study. Finally, it is important to recognize the cross-sectional nature of the data. As such, we were unable to assess changes in urinary cytokine/chemokine excretion over time, including the effect of intercurrent illness or changes in glycemic control on these factors.

In conclusion, hyperfiltration in adolescents with T1D is associated with higher levels of urinary cytokine/chemokine excretion, an effect that is in part dependent on ambient blood glucose levels. Future work is required to determine if high urinary cytokine/chemokine excretion rates are associated with early renal function decline or the onset of proteinuria. Future studies should also determine if suppression of urinary cytokines/chemokines with RAAS inhibition as observed in physiology studies can also be achieved in a clinical setting and if declines in these factors correlate with improved clinical outcomes [6].
